# *In vitro *anti-leishmanial and anti-fungal effects of new Sb^III ^carboxylates

**DOI:** 10.1186/2191-2858-1-2

**Published:** 2011-07-18

**Authors:** MI Khan, Saima Gul, Iqbal Hussain, Murad Ali Khan, Muhammad Ashfaq, Inayat-Ur-Rahman, Farman Ullah, Gulrez Fatima Durrani, Imam Bakhsh Baloch, Rubina Naz

**Affiliations:** 1 Department of Chemistry, Kohat University of Science & Technology, Kohat 26000, Khyber Pakhtunkhwa, Pakistan; 2 Department of Chemistry, The Islamia University of Bahawalpur, Bahawlpur, Punjab, Pakistan; 3 Faculty of Biological Sciences, Quaid-i-Azam University, Islamabad, Pakistan; 4 Institute of Pharmaceutical Sciences, Kohat University of Science and Technology, Kohat 26000, Khyber Pakhtunkhwa, Pakistan; 5 Department of Chemistry, Gomal University, Dera Ismail Khan, Khyber Pakhtunkhwa, Pakistan

**Keywords:** antimony(III) carboxylates, anti-leishmanial, anti-fungal

## Abstract

Ring opening of phthalic anhydride has been carried out in acetic acid with glycine, β-alanine, L-phenylalanine, and 4-aminobenzoic acid to yield, respectively, 2-{[(carboxymethyl)amino]carbonyl}benzoic acid (**I**), 2-{[(2-carboxyethyl)amino]carbonyl}benzoic acid (**II**), 2-{[(1-carboxy-2-phenylethyl)amino]carbonyl}benzoic acid (**III**), and 2-[(4-carboxyanilino)carbonyl]benzoic acid (**IV**). Compounds **I-IV **have been employed as ligands for Sb(III) center (complexes **V-VIII**) in aqueous medium. FTIR and ^1^H NMR spectra proved the deprotonation of carboxylic protons and coordination of imine group and thereby tridentate behaviour of the ligands as chelates. Elemental, MS, and TGA analytic data confirmed the structural hypothesis based on spectroscopic results. All the compounds have been assayed *in vitro *for anti-leishmanial and anti-fungal activities against five leishmanial strains *L. major *(JISH118), *L. major *(MHOM/PK/88/DESTO), *L. tropica *(K27), *L. infantum *(LEM3437), *L. mex mex *(LV4), and *L. donovani *(H43); and *Aspergillus Flavus, Aspergillus Fumigants, Aspergillus Niger*, and *Fusarium Solani*. Compound **VII **exhibited good anti-leishmanial as well as anti-fungal impacts comparable to reference drugs.

## Background

Trivalent antimony reagents are extensively consumed in industrial processes, e.g., in catalysis for the synthesis of polymers akin ethyleneterephthalate, with different brand names like Dacron^® ^and Mylar^®^. Similarly, antimony alkoxides have also been employed as precursors for the deposition of thin films of Sb_2_O_3 _and Sb_6_O_13 _[1-4]. The literature also revealed use of trivalent antimony compounds in fluorine chemistry and their suitability as solid electrolytes, piezoelectrics, and ferroelectrics [5,6]. On the other hand, the use of tri- and pentavalent antimony containing compounds as drugs for the treatment of leishmaniasis span more than 50 years; but little is known about the actual mechanisms of antimony toxicity and drug resistance [7,8]. Carboxylic group-containing compounds are versatile ligands to act as unidentate, bidentate, or bridging ligands; moreover, these also act as a spacer between Sb and other moieties [9-13]. All these facts prompted us to investigate the chemistry as well and biocidal effects of antimony^III ^complexes formed with ligands containing two carboxylic groups.

## Experimental

As received grade chemicals used during this study were procured from Sigma; the solvents were dried as reported [14]. C, H, and N analyses were carried out on a Yanaco high-speed CHN analyzer; antipyrene was used as a reference, while antimony was estimated according to the reported procedure [15]; melting points were recorded on Gallenkmp capillary melting point apparatus and are uncorrected. FTIR spectra of all the compounds were taken on Bruker FTIR spectrophotometer TENSOR27 using OPUS software in the range of 5000-400 cm^-1 ^(ZnSe). ^1^H and ^13^C NMR spectra in DMSO were recorded on a multinuclear Avance 300 and 75 MHz FT NMR spectrometer operating at room temperature, i.e., 25 C. Thermoanalytical measurements were carried out using a Perkin Elmer Thermogravimetric/differential thermal analyzer (YRIS Diamond TG-DTA High Temp. Vacu.) consuming variable heating rates between 0.5°C/min and 50°C/min. HR FAB-MS spectra were obtained from a double-focusing mass spectrometer Finnigan (MAT 112).

### Synthesis of ligands

Phthalic anhydride (5 g, 33.77 mM) was dissolved in acetic acid (100 mL), and a cold solution of amino acid (33.77 mM, i.e. 2.53 g, 3 g, 5.58 g, and 4.63 g of glycine, β-alanine, L-phenylalanine, and 4-aminobenzoic acid, respectively) in acetic acid (75 mL) was added to it. This mixture was stirred at room temperature for 3 hours resulting in white precipitate. The white precipitate was washed several times with cold water and recrystallized from water.

### Synthesis of I

Yield: 72%. C_10_H_9_NO_5_: Calcd. (%): C 53.82, H 4.06, and N 6.28; Found (%): C 53.27, H 3.86, N 6.01; FAB-MS (*m/z*) 224 (M + 1); IR ν 3293 (N-H), 1684 (C-N), 1592 (CO_2_)_as_, and 1353 (CO_2_)_s_, Δν (CO_2_): 239 cm^-1^. ^1^H NMR (DMSO-d6, 300 MHz) 12.8 (s, COOH), 12.1 (s, COOH), 8.31 (s, NH), 7.02-7.61 (Ar), and 3.62 (s, CH_2_). ^13^C NMR (DMSO-d6, 75 MHz) 174.7 (COOH), 170.2 (CONH), 168.9 (COOH), 107-138 (Ar), and 44.6 (CH_2_).

### Synthesis of II

Yield: 67%. C_11_H_11_NO_5_: Calcd. (%): C 55.70, H 4.67, N 5.90; Found (%): C 55.24, H 4.03, N 5.42. FAB-MS (*m/z*) 238 (M + 1). IR ν 3372 (N-H), 1670 (C-N), 1581 (CO_2_)_as_, 1345 (CO_2_)_s_, Δν (CO_2_): 236 cm^-1^. ^1^H NMR (DMSO-d6, 300 MHz) 12.7 (s, COOH), 12.5 (s, COOH), 8.39 (s, NH), 7.07-7.59, (m, Ar) 3.51 (t, CH_2 _*J*: 3.42), 2.33 (t, CH_2 _*J*: 4.1). ^13^C NMR (DMSO-d6, 75 MHz) 173.2 (COOH), 168.8 (COOH), 142.4 (CONH), 109-140 (Ar), 40.4 (CH_2_), 35.2 (CH_2_).

### Synthesis of III

Yield: 80%. C_17_H_15_NO_5_: Calcd. (%): C 65.17, H 4.83, N 4.47; Found (%): C 64.86, H 4.32, N 4.11. FAB-MS (*m/z*) 314 (M + 1). IR ν 3380 (N-H), 1686 (C-N), 1577 (CO_2_)_as_, 1361 (CO_2_)_s_, Δν (CO_2_): 216 cm^-1^. ^1^H NMR (DMSO-d6, 300 MHz) 12.6 (s, COOH), 12.2 (s, COOH), 8.43 (s, NH), 7.02-7.51 (m, Ar), 5.06 (q, CH, *J*: 8.8), 3.4 (d, CH_2_, *J*: 10.1). ^13^C NMR (DMSO-d6, 75 MHz) 171.4 (COOH), 170.0 (COOH), 144.1 (CONH), 111-138 (Ar), 61.2 (CH), 36.1 (CH_2_).

### Synthesis of IV

Yield: 70%. C_15_H_11_NO_5_: Calcd. (%): C 63.16, H 3.89, N 4.91; Found (%): C 63.02, H 3.43, N 4.60. FAB-MS (*m/z*) 286 (M + 1). IR ν 3388 (N-H), 1672 (C-N), 1566 (CO_2_)_as_, 1371 (CO_2_)_s_, Δν (CO_2_): 195 cm^-1^. ^1^H NMR (DMSO-d6, 300 MHz) 12.3 (s, COOH), 11.8 (s, COOH), 8.52 (s, NH), 7.13-8.33 (m, Ar). ^13^C NMR (DMSO-d6, 75 MHz) 176.7 (COOH), 165.4 (COOH), 148.2 (CONH), 120-136 (Ar).

### Synthesis of antimony complexes

Aqueous solution of SbCl_3 _was made by dissolving 0.5 g (2.19 mM) in 10 mL, and a few drops of dil. HCl were added; to this solution, equimolar amount of ligand 2.19 mM, i.e. 0.48 g, 0.52 g, 0.69 g, and 0.62 g, respectively, for **I-IV **dissolved in ethanol (20 mL). The mixture was stirred at room temperature for 15 min, for adjustment of pH, and one drop of ammonia was added which resulted in the formation of a precipitate. The precipitate was filtered and washed with warm 70% ethanol and recrystallized from water.

### Synthesis of V

Yield: 58%. C_10_H_7_ClNO_5_Sb: Calcd. (%): C 31.74, H 1.86, N 3.70, Sb 32.18; Found (%): C 31.21, H 1.45, N 3.39, Sb 31.80. FAB-MS (*m/z*) 377, 379 (M + 2). IR ν 3231 (N-H), 1655 (C-N), 1561 (CO_2_)_as_, 1320 (CO_2_)_s_, Δν (CO_2_): 241, 450 (N → Sb), 574 (O-Sb) cm^-1^. ^1^H NMR (DMSO-d6, 300 MHz) 8.24 (s, NH), 7.11-7.61 (m, Ar), 3.87 (s, CH_2_). ^13^C NMR (DMSO-d6, 75 MHz) 177.4 (CONH), 174.7 (COO), 170.2 (COO), 107-138 (Ar), 40.2 (CH_2_).

### Synthesis of VI

Yield: 58%. C_11_H_9_ClNO_5_Sb: Calcd. (%): C 33.67, H 2.31, N 3.57, Sb: 31.03; Found (%): C 33.28, H 2.08, N 3.19, Sb: 30.67. FAB-MS (*m/z*) 391, 393 (M + 2). IR ν 3265 (N-H), 1643 (C-N), 1551 (CO_2_)_as_, 1302 (CO_2_)_s_, Δν (CO_2_): 249, 442 (N → Sb), 582 (O-Sb) cm^-1^. ^1^H NMR (DMSO-d6, 300 MHz) 12.7 (s, COOH), 12.5 (s, COOH), 8.39 (s, NH), 7.07-7.59, (m, Ar) 3.51 (t, CH_2 _*J*: 3.42), 2.33 (t, CH_2 _*J*: 4.1). ^13^C NMR (DMSO-d6, 75 MHz) 181.4 (CONH), 170.0 (COO), 160.1 (COO), 122-142 (Ar), 33.3 (CH_2_NH), 27.1 (CH_2_).

### Synthesis of VII

Yield: 51%. C_17_H_13_ClNO_5_Sb: Calcd. (%): C 43.58, H 2.80, N 2.99, Sb 25.99; Found (%): C 43.20, H 2.50, N 2.67, Sb 25.34. FAB-MS (*m/z*) 467, 469 (M + 2). IR ν 3276 (N-H), 1666 (C-N), 1540 (CO_2_)_as_, 1311 (CO_2_)_s_, Δν (CO_2_): 229, 425 (N → Sb), 580 (O-Sb) cm^-1^. ^1^H NMR (DMSO-d6, 300 MHz) 8.24 (s, NH), 7.10-8.1 (m, Ar), 5.06 (t, CH, *J*: 9.7), 3.42 (d, CH_2_, *J*: 9.3). ^13^C NMR (DMSO-d6, 75 MHz) 180.6 (CONH), 174.0 (COO), 169.5 (COO), 125-136 (Ar), 66.8 (CH), 30.6 (CH_2_).

### Synthesis of VIII

Yield: 58%. C_15_H_9_ClNO_5_Sb: Calcd.(%): C 40.90, H 2.06, N 3.18, Sb 27.64; Found (%):C 40.71, H 1.89, N 2.91, Sb 27.22. FAB-MS (*m/z*) 439, 441 (M + 2). IR ν 3266 (N-H), 1678 (C-N), 1541 (CO_2_)_as_, 1336 (CO_2_)_s_, Δν (CO_2_): 137, 446 (N → Sb), 568 (O-Sb) cm^-1^. ^1^H NMR (DMSO-d6, 300 MHz) 8.16 (s, NH), 7.06-8.55 (m, Ar). ^13^C NMR (DMSO-d6, 75 MHz) 183.2 (CONH), 170.4 (COO), 172.8 (COO), 123-137 (Ar).

### Anti-leishmanial activity

Anti-leishmanial activity of the compound was carried out on the pre-established cultures of *L. major *(JISH118), *L. major *(MHOM/PK/88/DESTO), *L. tropica *(K27), *L. infantum *(LEM3437), *L. mex mex *(LV4) and *L. donovani *(H43). Parasites were cultured in medium M199 with 10% foetal bovine serum; 25 mM of HEPES, and 0.22 μg of penicillin and streptomycin, respectively at 24°C in an incubator. 1 mg of each test compound (**I-VIII**) was dissolved in 1 mL of water, ethanol, methanol and DMSO according to their solubilities. 1 mg of Amphotercin B was also dissolved in 1 mL of DMSO as reference drug. Parasites at log phase were centrifuged at 3000 rpm for 3 min. Parasites were diluted in fresh culture medium to a final density of 2 × 10^6 ^cells/mL. In 96-well plates, 180 μL of medium was added in different wells. 20 μL of the extracts was added in medium and serially diluted. 100 μL of parasite culture was added in all the wells. Four rows left for negative and positive controls: water, ethanol, methanol and DMSO, respectively, serially diluted in medium whereas positive control contained varying concentrations of standard antileishmanial compound, i.e. AmphotericinB. The plates were incubated for 72 h at 24°C. Results were analyzed through dose versus response by using nonlinear regression curve fit with Graphad Prims5.

### Anti-fungal activity

Agar tube dilution method was used for screening antifungal activities against *Aspergillus Flavus, Aspergillus Fumigants, Aspergillus Niger*, and *Fusarium Solani*. A sample of Media supplemented with DMSO and reference antifungal drugs was used as negative and positive control, respectively. Tubes were then incubated at 27°C for 4-7 days and examined twice weekly during incubation. Standard drug, Miconazole, used for the above stated fungi, growth in media containing sample under test were determined by linear growth (mm) measuring, and percent inhibition of growth was calculated with reference to negative control using formula.

## Results and discussion

**Figure 1:**
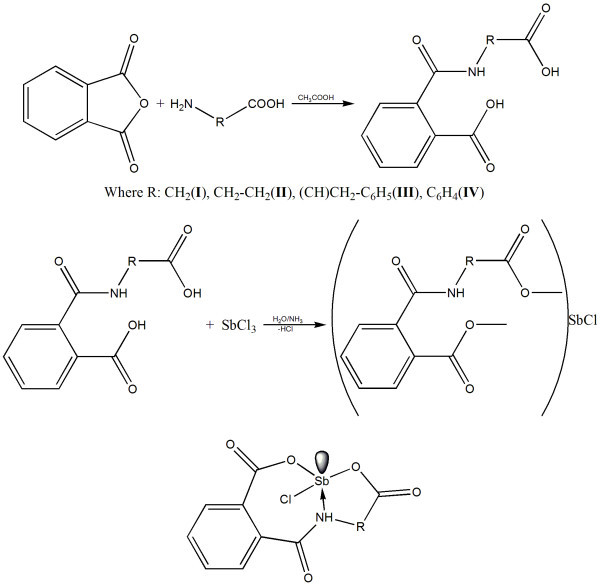
**Synthesis (I-VIII) and pseudotrigonal bipyramidal geometry**.

Ligands 2-{[(carboxymethyl)amino]carbonyl}benzoic acid (**I**), 2-{[(2-carboxyethyl)amino]carbonyl}benzoic acid (**II**), 2-{[(1-carboxy-2-phenylethyl)amino]carbonyl}benzoic acid (**III**), and 2-[(4-carboxyanilino)carbonyl]benzoic acid (**IV**), and the complexes (**V-VIII**), all of which were synthesized using a general procedure as shown in Figure 1. Analytic data for the complexes confirmed the equimolar stoichiometries thereby tridentate ligation (ONO) of **I-IV **towards Sb^III ^centre.

### FTIR spectra

Solid-state FTIR spectra were recorded in the spectral range of 4000-400 cm^-1^, and important vibrational frequencies were observed in this range. In the spectra of ligands (**I-IV**), characteristic broad band of carboxylic COOH functionality was observed in the range of 2800-3000 cm^-1^; OC-NH bond vibrated at 2600 cm^-1^; and aromatic C=C at 1500 cm^-1 ^[16]. Broad band observed for carboxylic group disappeared in the spectra of complexes indicating deprotonation of ligand. In the spectra of compounds **V-VIII**, appearance of new band of medium intensity around 430 cm^-1 ^indicated the coordination from N to antimony (O=C-NH → Sb) in pseudotrigonal bipyramidal arrangement (Figure 1) [17]. All the other bonds appeared at the same positions as in the spectra of the ligands ruling out coordination from carbonyl of phthalimido groups (Figure 1).

### Solution-state multinuclear NMR spectra

In the solution-state ^1^H and ^13^C NMR spectra of compounds (**V-VIII**), all the nuclei resonated at appropriate positions; in ^1^H NMR spectra, the disappearance of carboxylic protons confirmed deprotonation as observed in the FTIR spectra of ligands (**I-IV**). In addition, downfield shift of imine proton proved the coordinate linkage of imine group toward antimony center (-NH → Sb) [18]. Similarly, in ^13^C NMR spectra, carbonyl (C=O) adjacent to imine group resonated at downfield position compared with that of the ligands confirming coordination linakge of imine with antimony center; all these facts proved the 1:1 ligand to metal stoichiometry in pseudotrigonal bipyramidal geometry (Figure 1) [19-21]. Further, either of the carboxylic groups displayed different chemical shifts with carboxylic group attached to phenyl ring appeared slightly at high filed.

### MS & TGA analysis

**Figure 2:**
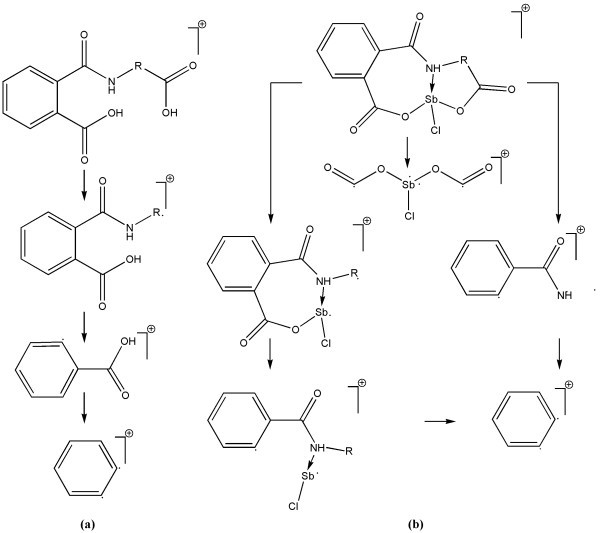
**MS fragmentation patterns**.

In the FAB MS spectra of complexes **VI-VIII**, base peak was observed at 245 *m/z *due to [O=C-O-(SbCl)-O-C=O]^+ ^fragment. Molecular ion peaks of very low intensity were observed with M + 2 peaks for isotopic ^123^Sb were also seen. Based on the data obtained, fragmentation patterns for ligands **I-IV **(Figure 2a) and complexes **V-VIII **(Figure 2b) have been proposed [20]. During the TGA analyses, heating rates were suitably controlled at 10°C/min under a nitrogen atmosphere, and the weight loss was measured ranging from ambient temperature up to 700°C. The weight losses for all the complexes were calculated for the corresponding temperature ranges and are shown in Table 1. The metal percentages left as metal oxide residues were compared with those determined by analytic metal content determination. Complexes **V-VIII **exhibited a three-stage decomposition pattern; as a first step, beginning of the weight loss occurred at 180, 178, 171, and 182 C, respectively, because of the escape of one C1 atom; next step of decomposition started at 280°C and extended up to 545°C corresponding to the loss of rest of the ligand's components and formation of metal oxide [22].

**Table 1:** Thermal analysis data of complexes V-VIII

	Loss of Cl			Oxide formation			% Metal	
Formula	Temp. range (°C)	Calculated	Found	Decomposition stage (°C)	Temperature (°C)	% Residue	Calculated	Found
C_10_H_7_ClNO_5_Sb	180-214	9.6	9.3	296-530	530	35	32.2	31.8
C_11_H_9_ClNO_5_Sb	178-207	9.2	8.8	300-485	485	66	31.0	30.7
C_17_H_13_ClNO_5_Sb	171-211	8.4	8.2	280-500	500	72	26.0	25.4
C_15_H_9_ClNO_5_Sb	182-220	8.9	8.5	308-550	550	70	27.6	26.3

All attempts employing different sets of conditions to obtain single crystals of the synthesized complexes suitable for XRD failed.

### Anti-leishmanial and anti-fungal activities

**Figure 3:**
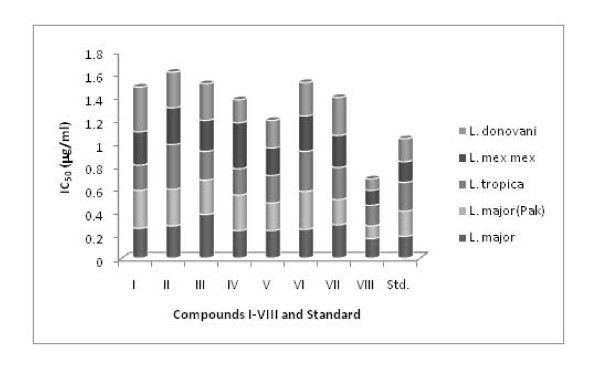
***In vitro *anti-leishmanial activity**.

All the compounds **I-VIII **were tested *in vitro *for their bioavailabilities against five leishmanial strains, i.e., *L. major *(JISH118), *L. major *(MHOM/PK/88/DESTO), *L. tropica *(K27), *L. infantum *(LEM3437), *L. mex mex *(LV4), and *L. donovani *(H43); and four fungi, viz., *Aspergillus Flavus, Aspergillus Fumigants, Aspergillus Niger*, and *Fusarium Solani *with one reference drug Amphotericin B, and the results are given in Tables 2 and 3, respectively. In general all the complexes (**V-VIII**) showed weaker activity compared to ligands (**I-IV**) and the reference drugs, but the complex **VIII **showed significant activity comparable to reference drugs. The activities (IC_50_) of all the compounds **I-VIII **together with AmphotericinB have been pictorially presented in Figure 3, and it is evident from the plot that the compound **VIII **exhibited significant activity. In complex **VIII**, the presence of bulkier R group, i.e., one benzyl moiety may be responsible for enhancement in drug uptake, thereby resulting significant activity [23,24].

**Table 2:** *In vitro *Anti-leishmanial effect (IC_50 _in μg/mL) of I-VIII and standard drug (AmphotericinB)

Leishmanial strain	Compound								
	I	II	III	IV	V	VI	VII	VIII	AmphotericinB
*L. major*	0.26	0.28	0.38	0.24	0.24	0.25	0.29	0.17	0.19
*L. major *(Pak)	0.33	0.32	0.30	0.31	0.24	0.33	0.22	0.11	0.22
*L. tropica*	0.22	0.39	0.25	0.23	0.24	0.35	0.28	0.18	0.25
*L. mex mex*	0.29	0.32	0.27	0.40	0.24	0.31	0.28	0.13	0.18
*L. donovani*	0.39	0.31	0.32	0.20	0.24	0.29	0.33	0.10	0.20

**Table 3:** *In vitro *Anti-fungal Effect of I-VIII and Standard Drug (Miconazole)

Fungi	Compound								
	I	II	III	IV	V	VI	VII	VIII	Miconazole
*Aspergillus flavus*	+	++	++	++	+	++	++	++++	+++
*Aspergillus Fumigants*	++	++	+	+++	++	++	+++	++++	+++
*Aspergillus Niger*	++	+	+	++	++	+	+++	++++	++++
*Fusarium Solani*	+++	++	++	+	++	+++	++	++++	+++

*Key*: +: No activity, ++: Low activity, +++: moderate activity, ++++: significant activity

## Conclusions

Antimony^III ^center in all the synthesized complexes is pseudotrigonal bipyramidal. Complex containing benzyl group displays noteworthy anti-leishmanial and anti-fungal effects. Proper understanding of exact relationship between structure and activity needs further research.

## Competing interests

The authors declare that they have no competing interests.
